# Influence of lipid bilayer on the structure of the muscle-type nicotinic acetylcholine receptor

**DOI:** 10.1073/pnas.2319913121

**Published:** 2024-04-29

**Authors:** Nigel Unwin

**Affiliations:** ^a^Medical Research Council Laboratory of Molecular Biology, Cambridge CB2 0QH, United Kingdom

**Keywords:** tubular vesicle, submembrane helix, fast synaptic transmission, cryo-EM

## Abstract

The muscle-type nicotinic acetylcholine receptor is a transmitter-gated ion channel residing in the plasma membrane of electrocytes and striated muscle cells. It is present predominantly at synaptic junctions, where it effects rapid depolarization of the postsynaptic membrane in response to acetylcholine released into the synaptic cleft. Previously, cryo-EM of intact membrane from *Torpedo* revealed that the lipid bilayer surrounding the junctional receptor has a uniquely asymmetric and ordered structure, due to a high concentration of cholesterol. It is now shown that this special lipid environment influences the transmembrane (TM) folding of the protein. All five submembrane MX helices of the membrane-intact junctional receptor align parallel to the surface of the cholesterol-ordered lipids in the inner leaflet of the bilayer; also, the TM helices in the outer leaflet are splayed apart. However in the structure obtained from the same protein after extraction and incorporation in nanodiscs, the MX helices do not align to a planar surface, and the TM helices arrange compactly in the outer leaflet. Realignment of the MX helices of the nanodisc-solved structure to a planar surface converts their adjoining TM helices into an obligatory splayed configuration, characteristic of the junctional receptor. Thus, the form of the receptor sustained by the special lipid environment of the synaptic junction is the one that mediates fast synaptic transmission; whereas, the nanodisc-embedded protein may be like the extrajunctional form, existing in a disordered lipid environment.

## Significance

The nicotinic acetylcholine receptor at the nerve-muscle synapse resides in an asymmetric cholesterol-rich membrane, the inner leaflet of which consists largely of ordered lipid assemblies due to a high cholesterol concentration. Cryo-EM of intact synaptic membrane shows that the peripheral submembrane α-helices MX of the receptor align parallel to the surface of these cholesterol-ordered lipids. However, in structures obtained from the same protein after detergent extraction, the MX helices adopt an alternative nonplanar configuration, which is coupled to a more compact arrangement of the transmembrane helices. Thus, the special lipid environment of the synaptic junction is required to sustain the normal physiological form of the receptor involved in fast synaptic transmission, a property inferred from electrophysiological evidence obtained decades ago.

The postsynaptic membrane at the synaptic junctions of electrocytes and striated muscle cells comprises a cholesterol-rich phospholipid bilayer, most densely populated by a single membrane-spanning protein, the nicotinic acetylcholine receptor. This well-characterized heteropentameric ion channel forms regular closely packed arrays, an organization thought to maximize the speed and magnitude of depolarization of the membrane in response to acetylcholine released into the synaptic cleft (1). Surrounding each receptor in these arrays is an asymmetric lipid bilayer, the inner (cytoplasmic) leaflet of which consists largely of ordered lipid assemblies formed as a result of cholesterol being present in saturating amounts (2). This lipid environment, unique to the synaptic junction, contrasts with that of other regions of the cell membrane, where lower cholesterol levels (3) would lead to a more disordered and mobile lipid setting.

The question therefore arises: Is the structure of the receptor, influenced by the junctional bilayer, any different from that existing in other regions of the cell membrane? In fact, several decades ago, electrophysiologists working with normal and denervated frog muscle fibers discovered that there are two populations of muscle-type acetylcholine receptors: junctional receptors, i.e., those located within the synaptic junction; and extrajunctional receptors, present in more distant regions of the cell membrane (4–6). The junctional receptors exhibited more rapid gating kinetics and were two to three times more conductive (4) than those in extrajunctional regions.

These experiments hinted that the differences between the two kinds of receptors might lie in disparate lipid environments (5). It is shown here that the structure of the membrane-intact junctional receptor from *Torpedo* differs significantly from that of the extracted protein incorporated in nanodiscs (7, 8), where the disordered lipid environment would resemble more closely the environment of the extrajunctional form. A key element responsible for the difference is the submembrane helix MX of the receptor, which establishes a coplanar alignment with the surface of the cholesterol-ordered leaflet of the junctional bilayer. Thus, the special lipid environment of the synaptic junction plays an essential role in maintaining the form of the receptor that mediates fast synaptic transmission.

## Results

**Fig. 1.**
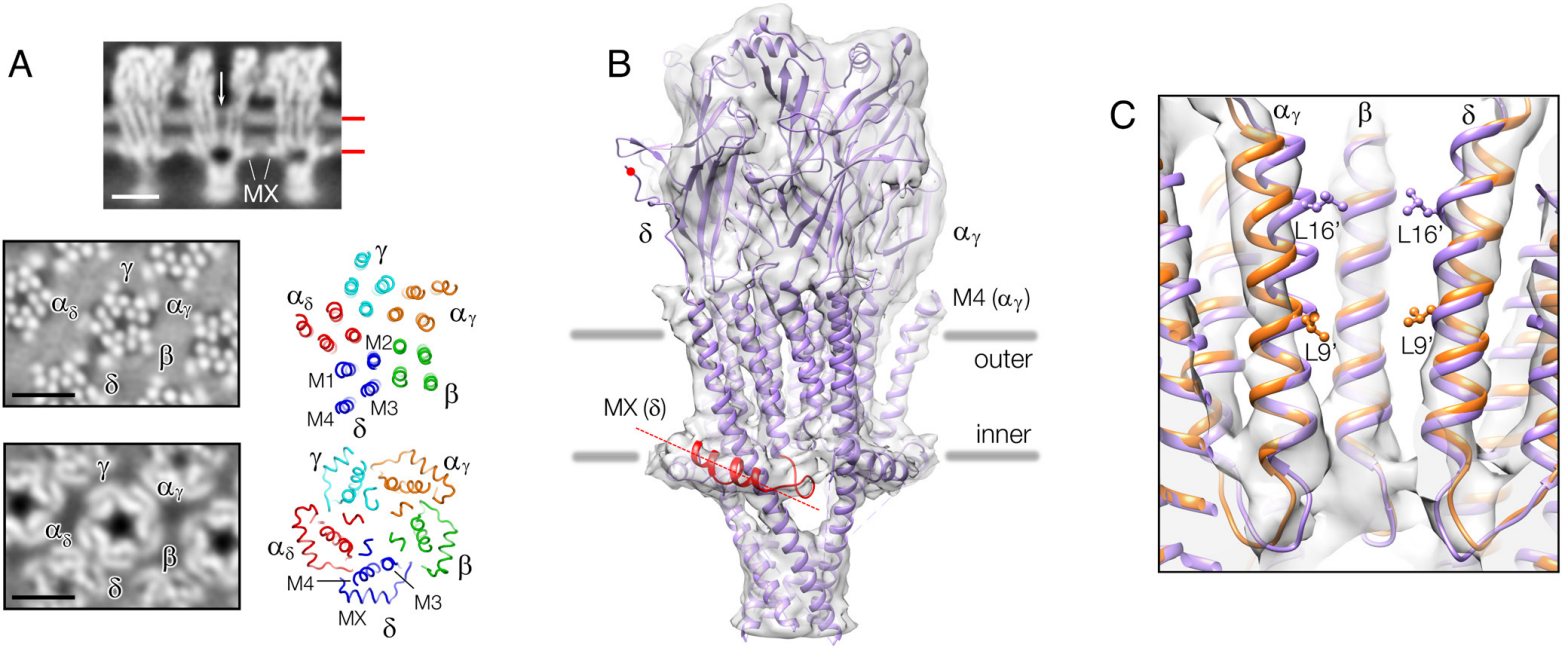
Overview and structure of the acetylcholine receptor in tubular vesicles. (*A*) Profile view intersecting the tapered pore (arrow, *Top*), from the unmasked density map [(−16,6) tube; *SI Appendix*, Fig. S1], and in-plane slices passing through the outer phospholipid headgroups (*Middle*) and MX helices (*Bottom*) at levels indicated by red bars; the tube axis is horizontal; Scale bar, 50 Å; 10-Å thick slabs from the structure determined here are aligned with details of the central receptor and labeled to indicate subunit and α-helical assignments. (*B*) Nanodisc-model (PDB ID code: 7SMM) superimposed on the 4.7 Å density map of a single receptor; MX of the δ subunit and the δ-δ disulfide bridge-forming cysteine are highlighted in red. (*C*) Slab encompassing the M2 helices of α_γ_, β, and δ lining the central membrane-spanning pore. The junctional structure (gold), obtained by fitting the nanodisc-model (purple) to the density map, has a less compact TM helical arrangement, leading to a less constrictive gate configuration. Rings of leucines at the 16′ and at the 9′ positions form barriers to ion permeation across the membrane.

The membrane-spanning portion of the acetylcholine receptor has four TM helices (M1–M4) and one submembrane helix, MX, comprising each of its five subunits (α_γ_, β, δ, α_δ_, γ). The junctional form of this protein, analyzed by cryo-EM of postsynaptic-membrane-derived tubular vesicles (*Methods*), has a tapered pore (Fig. 1 *A*, *Top*) framed by a splayed arrangement of TM helices (Fig. 1 *A*, *Middle*) and a symmetrical rim of MX helices lying flat against the inner (cytoplasmic) bilayer surface (Fig. 1 *A*, *Bottom*). In addition, time-resolved experiments on the tubular vesicles have shown that loop C of the α_γ_ subunit closes around the binding site, and that the pore widens, on brief exposure to acetylcholine (9), indicating that the (unreacted) junctional receptor is in a closed (resting) and activatable state.

The present study is based on a 4.7 Å density map of the junctional receptor (Fig. 1*B*) obtained from two helical families of tube (*Methods* and *SI Appendix*, Figs. S1 and S2 and Table S1). The densities in the extracellular and intracellular domains are largely consistent with nanodisc-solved structures of the closed channel (Fig. 1*B*) (7, 8), although there is mismatch in the case of the δ subunit. This is to be expected, since the disulfide bridge linking the δ subunits of neighboring receptors in the native membrane (10) is absent in the nanodisc-embedded protein, leaving the unbonded cysteines far from the membrane surface (red dot; Fig. 1*B*).

More significantly, the membrane-spanning portions of the junctional and nanodisc-solved structures show differences that evidently are dependent on the alternative natural or artificial lipid environments in which the protein is embedded. Most obvious at lower resolution, the junctional receptor has a more splayed arrangement of TM helices in the outer leaflet of the bilayer (11).

### Membrane-spanning Pore.

To characterize further the junctional receptor in relation to the nanodisc form, a model of the junctional form was obtained by fitting the nanodisc-solved structure to the density map, using real-space refinement (*Methods* and *SI Appendix*, Fig. S3). Fig. 1*C* compares details around the pore, where the encircling pore-lining M2 helices come close together to form the gate of the channel. In the junctional protein (gold), the upper portion of the pore is tapered (as in Fig. 1 *A*, *Top*) due to the fact that the M2 helices separate from one another in the outer leaflet of the bilayer. In the nanodisc-embedded protein (purple), this does not happen and so its pore, especially in the upper portion, is more constricted (see also *SI Appendix*, Fig. S4*A*). Biochemical studies on isolated *Torpedo* postsynaptic membranes have identified the conserved leucine at the 9′ position (Fig. 1*C*) as a major gate-forming residue (12), in agreement with the narrowing of the junctional pore at this point. The whole 9′ to 16′ portion of the pore in the nanodisc-embedded protein apparently creates a more extensive gate (7, 8).

Hence, the pore-lining (and other) TM helices have alternative configurations representing the closed (or resting) state of the channel, depending on whether the protein is in junctional or nanodisc lipid environments.

### Helix MX at the Bilayer Surface.

**Fig. 2.**
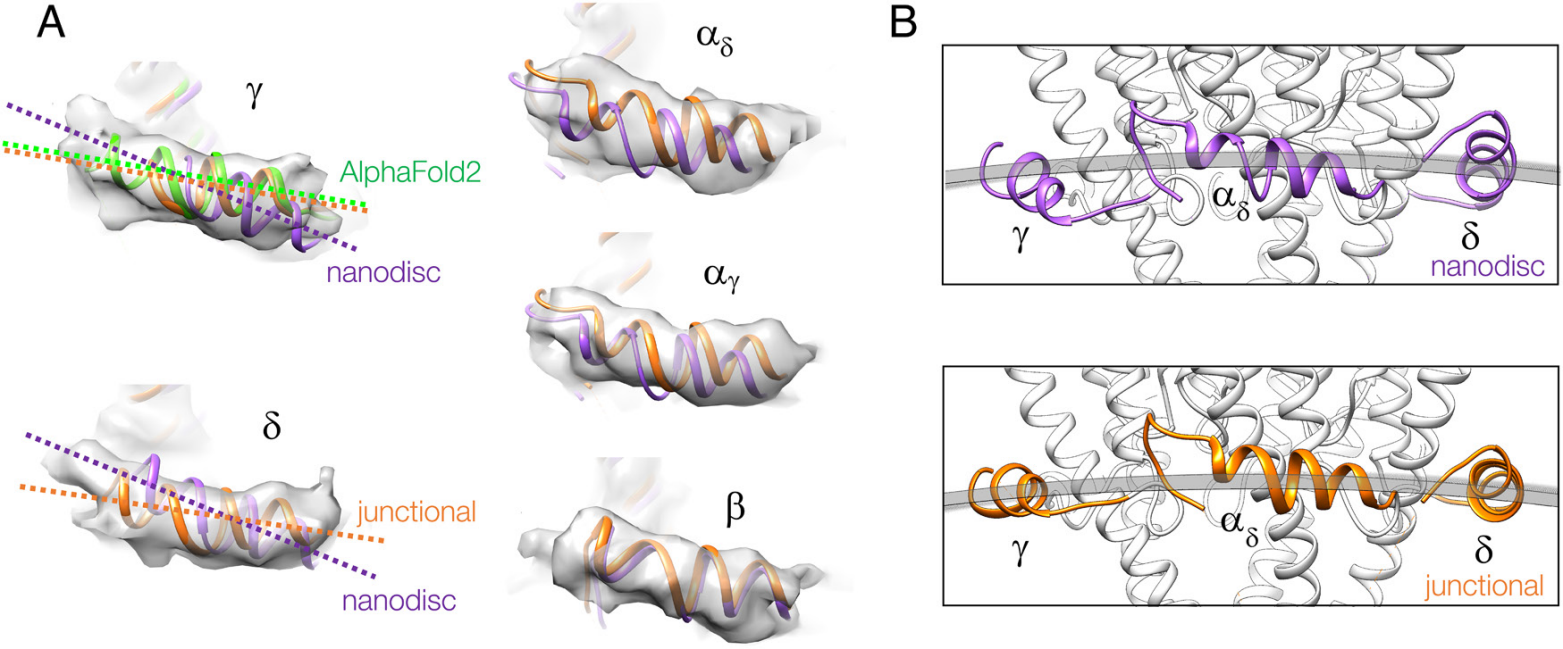
Different orientations of MX in the two receptor forms. (*A*) Experimental densities and superimposed MX helix structures. MX(γ) and MX(δ) of the nanodisc-solved structure (purple) are tilted by ~13° away from their orientations in the junctional structure (gold). MX(γ) from the AlphaFold2 structure of the γ subunit, after (flexible) fitting to the densities, is shown in green. (*B*) Cross-sections encompassing MX(γ), MX(α_δ_), and MX(δ) intersected by a cylindrical arc having a curvature equal to that of the tubular membrane. Only the junctional MX helices (gold) align closely to the cylindrical arc.

The alternative pore-lining M2 configurations, it will be shown, relate to the way the MX helices align with the bilayer surface. Fig. 2*A* compares the densities and poses of the five individual MX helices in the two forms. The densities corresponding to the junctional form show all five helices to be about equally resolved and equal in length. In comparison, the nanodisc MX(γ) is foreshortened (and is better represented by the AlphaFold2 version of this subunit) (13, 14). Also, the nanodisc MX(γ) and MX(δ) are both much more inclined (by ~13°; see also Fig. 1*B*) than their junctional counterparts.

These discrepancies, and lesser ones in MX(α_δ_) and MX(α_γ_), give rise to distinct submembrane structures when the junctional and nanodisc forms are viewed in cross-section (Fig. 2*B*). Only the junctional MX helices align closely with a cylindrical arc of curvature equivalent to that of the bilayer surface and so have a near-planar arrangement.

**Fig. 3.**
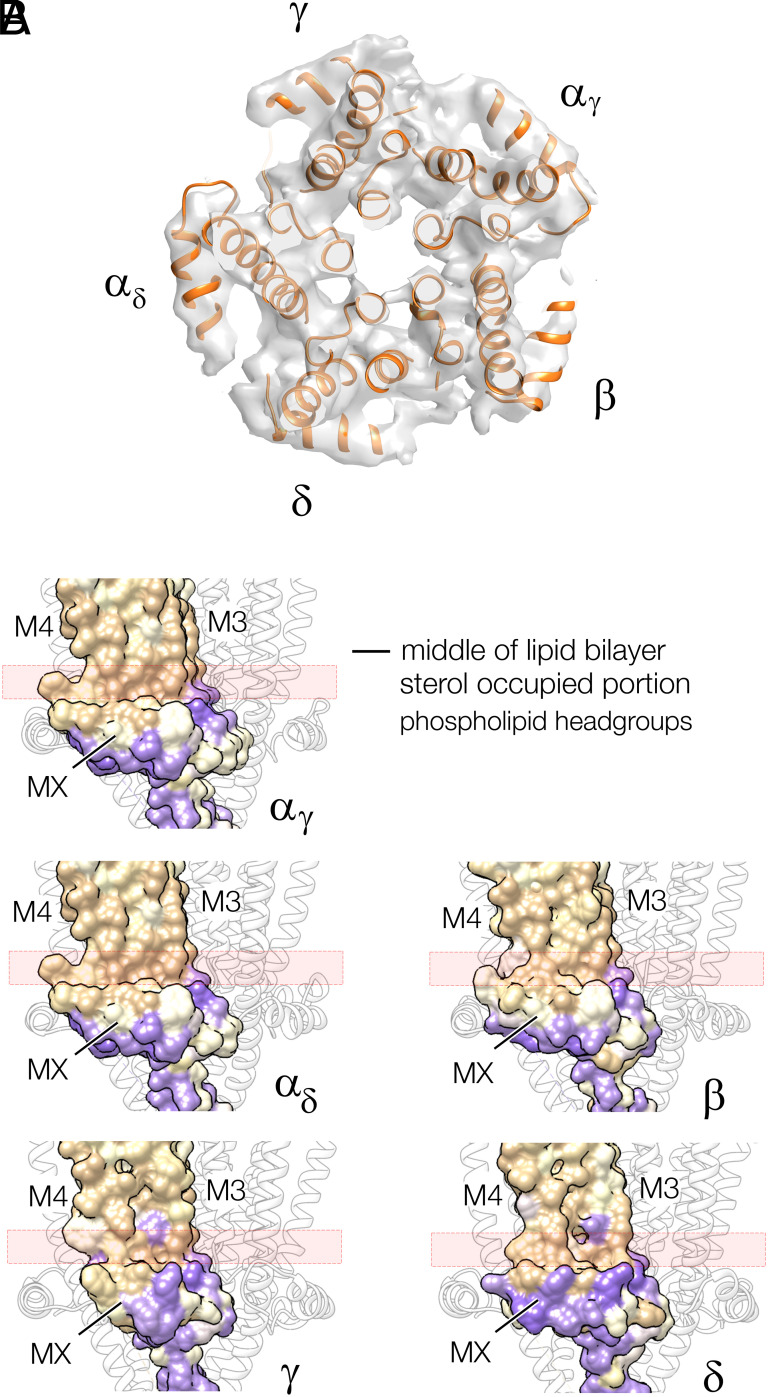
Near-planar configuration of MX helices framing the junctional receptor. (*A*) Lipid-exposed faces of the MX helices. (*B*) Hydrophobicity surface representation of MX and adjoining M4 and M3 helices in the inner leaflet of the bilayer (tan, hydrophobic; purple, polar). The MX helices (viewed from the side) present a polar face toward the zwitterionic phospholipid headgroups, and a flat hydrophobic face toward the hydrophobic core of the bilayer. The pink rectangle overlying this flat surface indicates the extent of the sterol-occupied portion of the bilayer (2).

Fig. 3*A* shows the lipid-exposed faces of the junctional MX helices, viewed from the membrane interior. The fact that these helices both lie parallel to and penetrate the headgroup region of the bilayer (Fig. 1 *A*, *Top*) (2) implies that they engage directly with the cholesterol-ordered lipids: a possibility that does not exist in the nanodisc environment. Interestingly, the hydrophobic amino acids along MX project near-equally toward the lipid core in all five subunits (Fig. 3*B*), while the sterol groups potentially create a rather uniform surface, because of their ordering. Hence, it is matching flatness as well as hydrophobicity that may bring these two components together.

### Coupling of MX with Adjoining TM Helices.

**Fig. 4.**
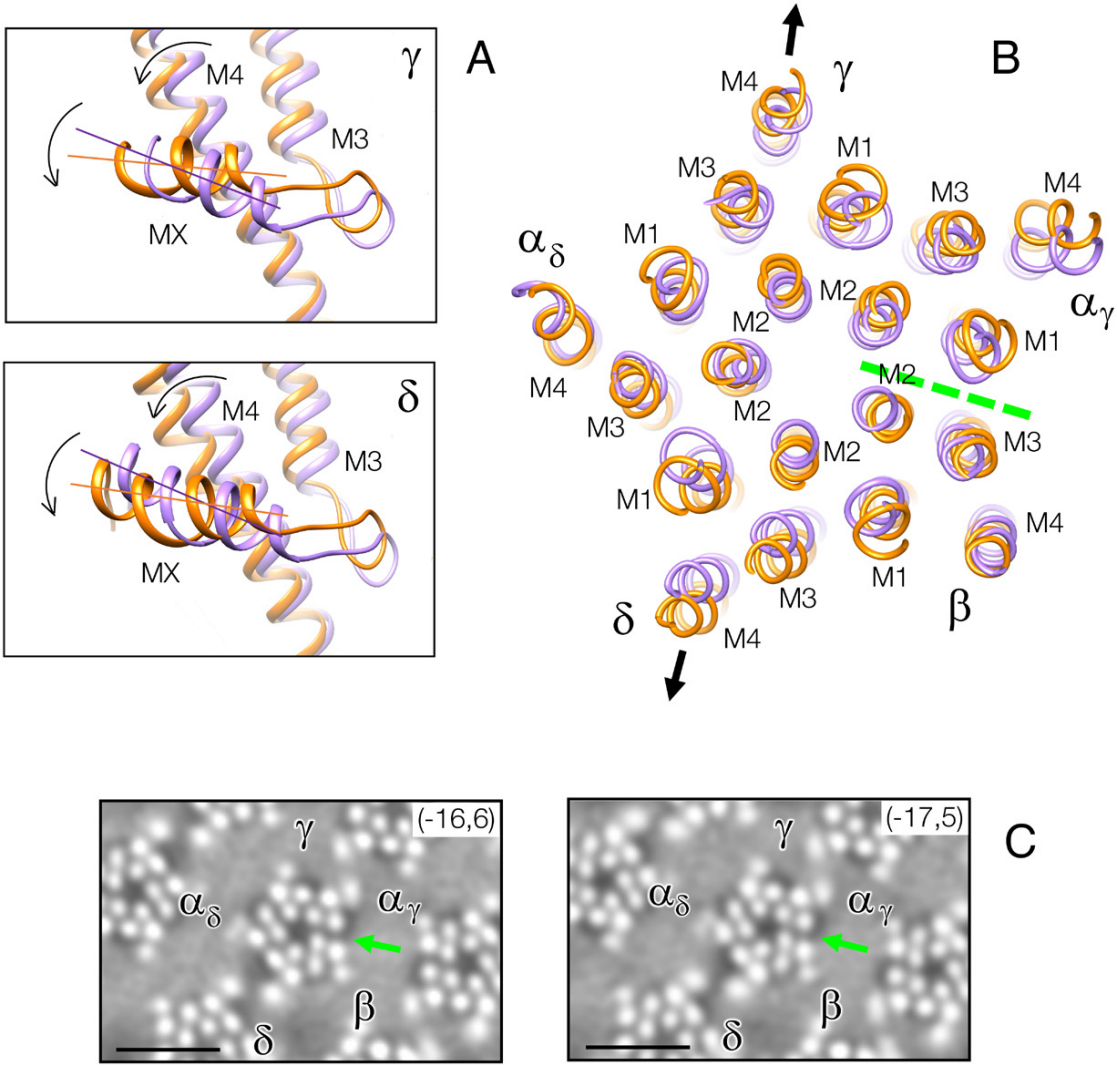
Realignment of MX to the bilayer surface enforces rearrangement of the TM helices. (*A*) Slabs through γ and δ subunits encompassing MX, M4, and M3 of the nanodisc-solved structure before (purple) and after (gold) fitting to the densities: reorientation of MX and tilting outward of M4/M3 (mainly M4) are coupled by related rotations (arrows). (*B*) Slab encompassing the phospholipid headgroup region in the outer leaflet. The helices of the junctional receptor (gold) are more splayed than those of the nanodisc-solved structure (purple) due to outward tilt of M4/M3 of γ and δ (arrows), together with accompanying adjustments of the other TM helices. The α_γ_/β interface (broken line) widens the most as a result of these adjustments. (*C*) The widened α_γ_/β interface (arrow) viewed in slices through the outer phospholipid headgroup region in the unmasked density maps; dark patches in the lipid regions next to the TM helices, in the vicinity of the interfaces, signify the presence of underlying cholesterol (11). (Scale bar, 50 Å.)

MX and the MX–M3 connecting loop make extensive hydrophobic contacts with the TM helices, M4 and M3. Therefore, the angular changes needed to bring the tilted MX (γ) and MX(δ) into alignment with the bilayer surface inevitably implicate M4 and M3. Comparison of the two receptor forms in this region (Fig. 4*A*) shows that M4 and, to a lesser extent, M3, would indeed undergo rotations in the same sense as MX. These rotations would account qualitatively for the 2 Å to 3 Å outward displacements of M4 and M3, in both subunits, in the outer phospholipid headgroup region of the bilayer (arrows, Fig. 4*B* and *SI Appendix*, Fig. S4*B*).

Analyzed as a global rearrangement (*SI Appendix*, Fig. S5), the adjacent M1 and M2 helices in γ and δ also move outward to accommodate the changes in M4 and M3 (Fig. 4*B*). In addition, the other subunits readjust, mainly through rigid-body displacements, to maintain favored interactions with their neighbors. For example, α_γ_ is displaced outward together with γ in the headgroup region (Fig. 4*B*), widening by ~2 Å the interface between α_γ_ and its other neighbor, the β subunit (Fig. 4*C* and *SI Appendix*, Fig. S4*C*). Likewise, but to lesser extents, the other subunit–subunit interfaces are widened at this level in the structure. These small concerted changes together account for the observed splayed TM helical arrangement of the junctional receptor. Cholesterol–protein interaction in the outer leaflet (Fig. 4*C*), as well as in the inner leaflet, may help stabilize this form.

In summary, the coupling that exists between the sub- and transmembrane elements is central to the relationship between the two receptor forms. Particularly important is the tight coupling between MX and the most lipid-exposed TM helix, M4 (15, 16). Apparently, the two receptor forms are interconvertible, determined by this coupling and by the composition and state of the surrounding lipids. One would therefore expect adjustments in the disposition of M4 brought about by the presence or absence of specific lipids (16) to affect also the disposition of MX.

## Discussion

The results above support the long-held notion that the special lipid environment of the synaptic junction is required to maintain the form of the acetylcholine receptor that mediates fast synaptic transmission. The junctional form is distinguished by having all five of its submembrane MX helices lying flat against the cholesterol-ordered bilayer surface (Fig. 3). The nanodisc-solved structure does not share this property, and so may be like the form of receptor present in other regions of the cell membrane, where the lipids are more disordered.

**Fig. 5.**
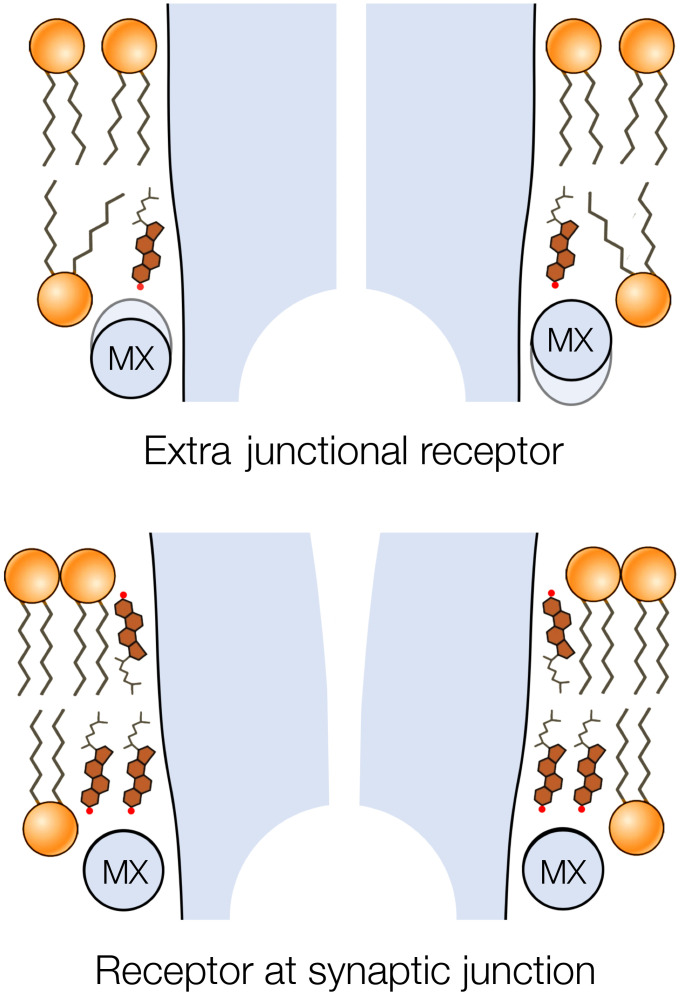
Influence of ordered lipid environment on transmembrane structure. It is suggested that helices MX of the receptor may adopt tilted poses, similar to those in nanodisc-solved structures, in the disordered extrajunctional regions of the cell membrane. However, the inner leaflet of the postsynaptic membrane introduces an alternative flat interaction surface due to cholesterol-induced ordering of the lipids. Realignment of the MX helices to engage in parallel with this surface forces the coupled TM helices to splay apart, creating a tapered pore.

Fig. 5 sketches how receptors in extrajunctional regions, assumed similar to the nanodisc-embedded receptor, might convert to the junctional form, based on the details found here. The MX helices, due to their hydrophobic and flat lipid-facing surfaces, are drawn into alignment with the cholesterol-ordered bilayer, producing an obligatory readjustment of the TM helices to which they are coupled. This readjustment confers a splayed α-helical conformation, which is the one the receptor requires in order to perform optimally at the synapse.

As already described, the receptor in this study is analyzed as an integral component of the electrocyte cell membrane. It is the sole example in the acetylcholine receptor family of ion channels wherein the protein is evaluated in a native membrane setting, rather than in detergent or after reconstitution into nanodiscs. Furthermore, the structure of this protein is unique among the fourteen muscle-type receptor nanodisc structures so far solved (7, 8, 17–19) in having all MX helices aligned to a planar surface, as if engaging in a specific way with a lipid bilayer. This raises the question of whether the limited dimensions and artificial lipid environment of the nanodisc (20–22) really can sustain a protein conformation in which properties of a cell membrane, such as lipid asymmetry and cholesterol-induced ordering, may be pivotal to the establishment of the precise physiological form. The ABC transporter MsbA exhibits widely different conformations depending on whether it is observed in nanodiscs or analyzed in the context of the native cell membrane (23), underlining the importance of interpreting with caution the structures of proteins extracted from the lipid domains in which they function.

Fortunately, advances continue to be made in cryo-EM techniques, and it might soon become feasible to assess the nanodisc-solved structures by single-particle analysis of the same proteins in cell-derived membrane vesicles (24), or by in situ cryoelectron tomography (25). Even a modest resolution, like that attained here, should be sufficient to test whether nanodisc reconstitution provides a means to recapitulate accurately the conformations of the receptor as they exist in the cell membrane.

## Conclusions

The structure of the acetylcholine receptor at the synaptic junction differs significantly from the structure of the same protein in a nonjunctional lipid setting.

A key physiological role of the submembrane helix MX of the receptor is to sense the cholesterol-ordered lipid environment of the synaptic junction.

The MX helix, in engaging with the cholesterol-ordered lipids, causes the coupled TM helices to splay apart, creating the conformation the receptor requires to perform optimally at the synapse.

## Methods

### Specimen Preparation.

Postsynaptic membranes were isolated from fresh *Torpedo marmorata* electric organ and incubated in low-salt buffer (100 mM sodium cacodylate, 1 mM calcium chloride, pH 7) to form tubular vesicles (26). Acetylcholine receptors in the tubular vesicles arrange on a helical surface lattice, with the same local organization as they have in situ at the synaptic junctions of *Torpedo* electrocytes and at the frog neuromuscular junction (1, 27, 28).

### Cryo-EM and Structure Analysis.

Aliquots of the tube-containing solution were applied to perforated EM grids and plunge-frozen in liquid ethane. Micrographs of straight ice-embedded tubes were recorded at 300 kV on a FEI Titan Krios electron microscope (Thermo Fisher Scientific), using a Falcon 3 direct-electron detector operating in integrating mode. The total dose was 40 e Å^−2^, fractionated from 79 frames. Underfocus values ranged from 1.2 to 2.8 μm. The calibrated pixel size on the specimen was 1.34 Å. Micrograph frame stacks were drift-corrected and dose-weighted using *MotionCor2* (29). Local contrast transfer functions were estimated from the aligned, non-dose-weighted micrographs using *Gctf* (30).

All subsequent image processing steps were performed in *RELION* (31, 32). Two helical families of tube [(−16,6) and (−17,5) (33)] were analyzed, following initial *FFT*-based selection of the micrographs with *Ximdisp* (34). Tubes from the selected micrographs were divided into overlapping segments using a box size of 1,024 pixels and an interbox spacing of 80 pixels. The image processing workflow is summarized in *SI Appendix*, Fig. S1. With each family, four rounds of two-dimensional classification, applied to the extracted segments, yielded ~80% of sufficient quality for further processing. The three-dimensional classification was conducted in two rounds to obtain class averages characterized by distinct values for the helical parameters (twist and rise) and for tube radius. The best class averages obtained in this way [15 and 19 for the (−16,6) and (−17,5) families, respectively] were refined, using average values for the helical twist and rise, to yield the family-specific reconstructions (*SI Appendix*, Table S1). A value for the regularization parameter T = 10 was applied throughout.

For each helical family, densities corresponding to single receptors were cut out at radially equivalent coordinates from the individual reconstructions, using a soft spherical mask, and averaged (*SI Appendix*, Fig. S2*A*). The final “single particle” density map was obtained by averaging the reconstructions from each helical family, taking into account that their lattices are rotated relative to each other by 3.6°. A negative B factor (B = −350 Å^−2^) was applied to sharpen this map (35). Fourier shell correlation indicated resolutions of 5.0 Å for the two family-specific density maps and 4.7 Å for the average (*SI Appendix*, Fig. S2*B*).

Fitting of the atomic model to the 4.7 Å final density map was done by maximizing the correlation between the experimental densities and the densities computed from the model, using *DireX* (36). The extracellular domain of the receptor was omitted from these calculations. Refinement parameters were chosen to minimize changes to the original secondary structure. Pairwise comparison of subunits in the fitted structure with those in the atomic model yielded root-mean-square deviations (Cα atoms) of 1.7 to 2.8 Å, when evaluated over the transmembrane region, including MX (*SI Appendix,* Fig. S5).

All structural depictions in this study are based on a 2.5 Å model (PDB ID code: 7SMM) of the unliganded nicotinic acetylcholine receptor obtained by detergent extraction from *Torpedo californica* electric organ, followed by reconstitution into nanodiscs (7). Other sub-3 Å structures of the same protein in the absence of ligand (PDB ID codes: 7SMQ and 7QKO) (7, 8) are almost identical. Alignment of models to the map and preparation of the structural figures were done in *Chimera* (37) and *PyMol* (38).

## Supplementary Material

Appendix 01 (PDF)

## Data Availability

The final cryo-EM density map and the atomic coordinates of the fitted region of the membrane-intact junctional receptor have been deposited in the Electron Microscopy Data Bank and the Protein Data Bank under accession codes: EMD-18596 (39), 8QQD (40), respectively. All other data are included in the manuscript and/or *SI Appendix*.
