# Supplementary material for: Influence of lipid bilayer on the structure of the muscle-type nicotinic acetylcholine receptor
Source: Proc Natl Acad Sci U S A. 2024 Apr 29;121(19):e2319913121. doi: 10.1073/pnas.2319913121 (PMC11087746; doi:10.1073/pnas.2319913121)
Supplement: Supplementary file 1 — Appendix 01 (PDF) [file pnas.2319913121.sapp.pdf]

Supplementary Information for

Influence of lipid bilayer on the structure of the muscle-type nicotinic acetylcholine receptor

Nigel Unwin

Corresponding Author: Nigel Unwin
Email: unwin@mrc-lmb.cam.ac.uk

This PDF file includes:

Figures S1 to S5
Table S1
SI References

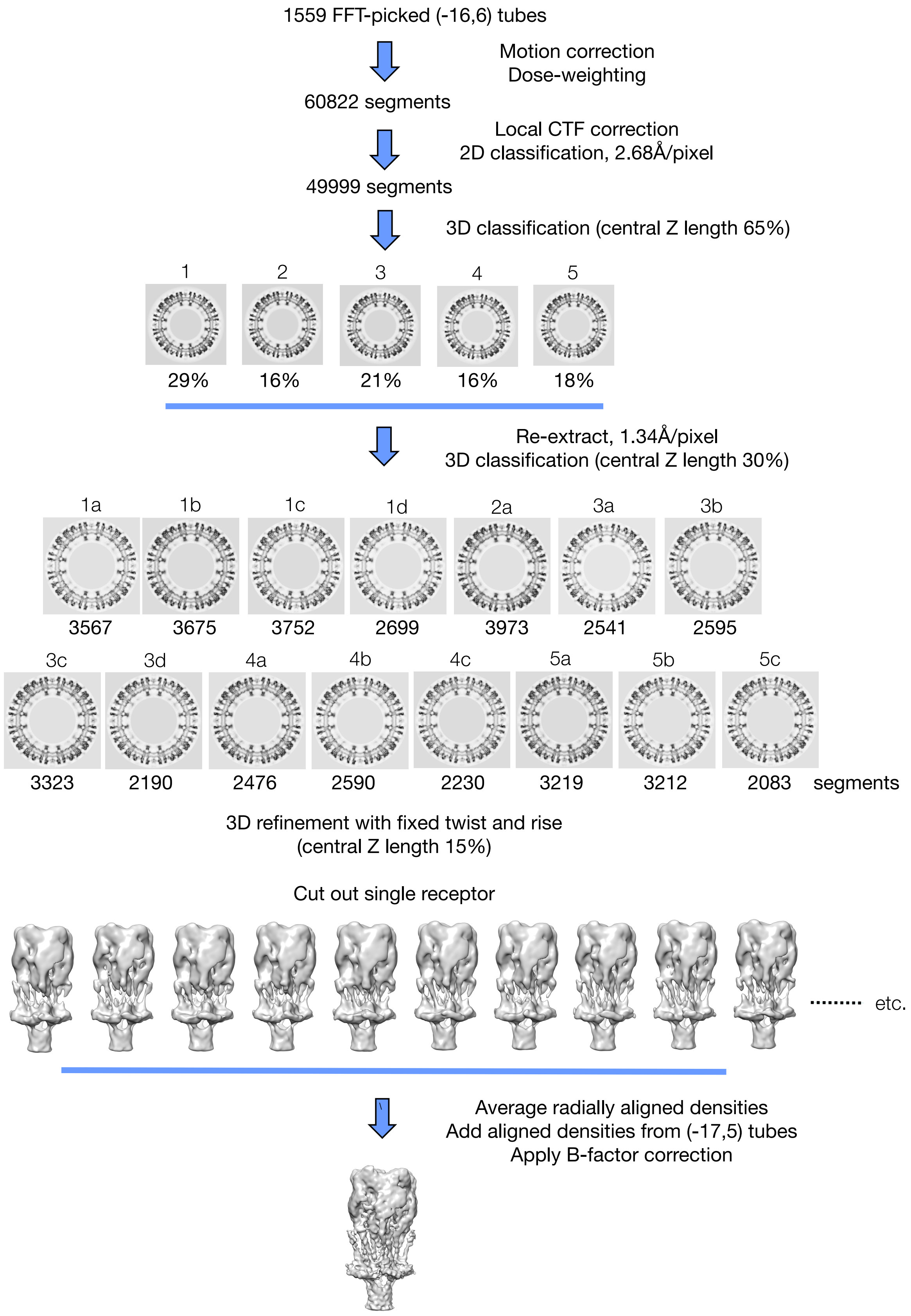

Fig. S1. Image processing workflow to determine the structure of a single receptor, illustrated with data from (- 16,6) tubes. The same steps were applied to the (-17,5) tubes, using 2486 micrographs (19 class averages), and the densities from the two helical families were averaged. See Table S1 for further details.

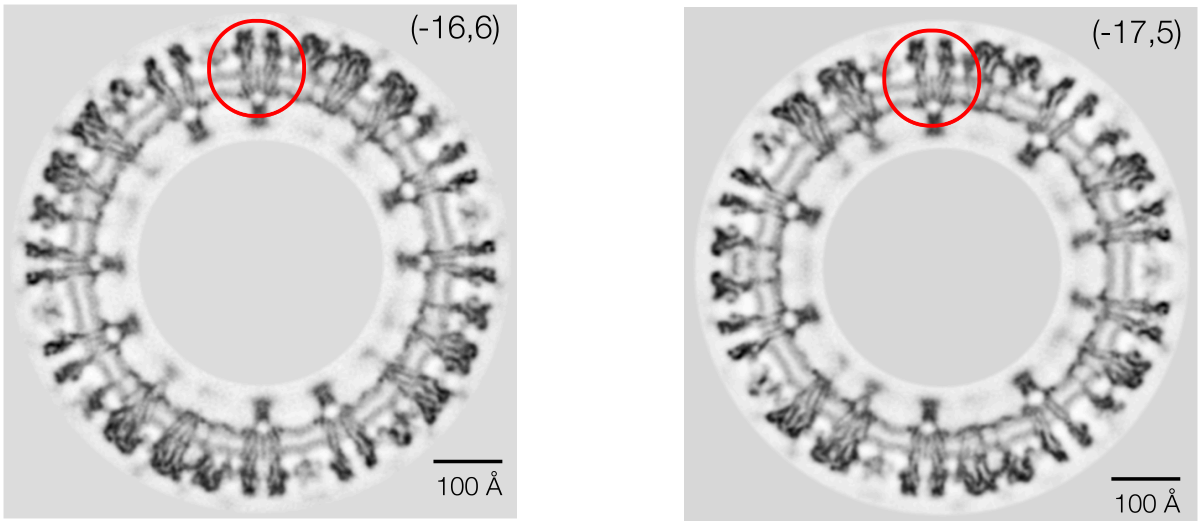

A

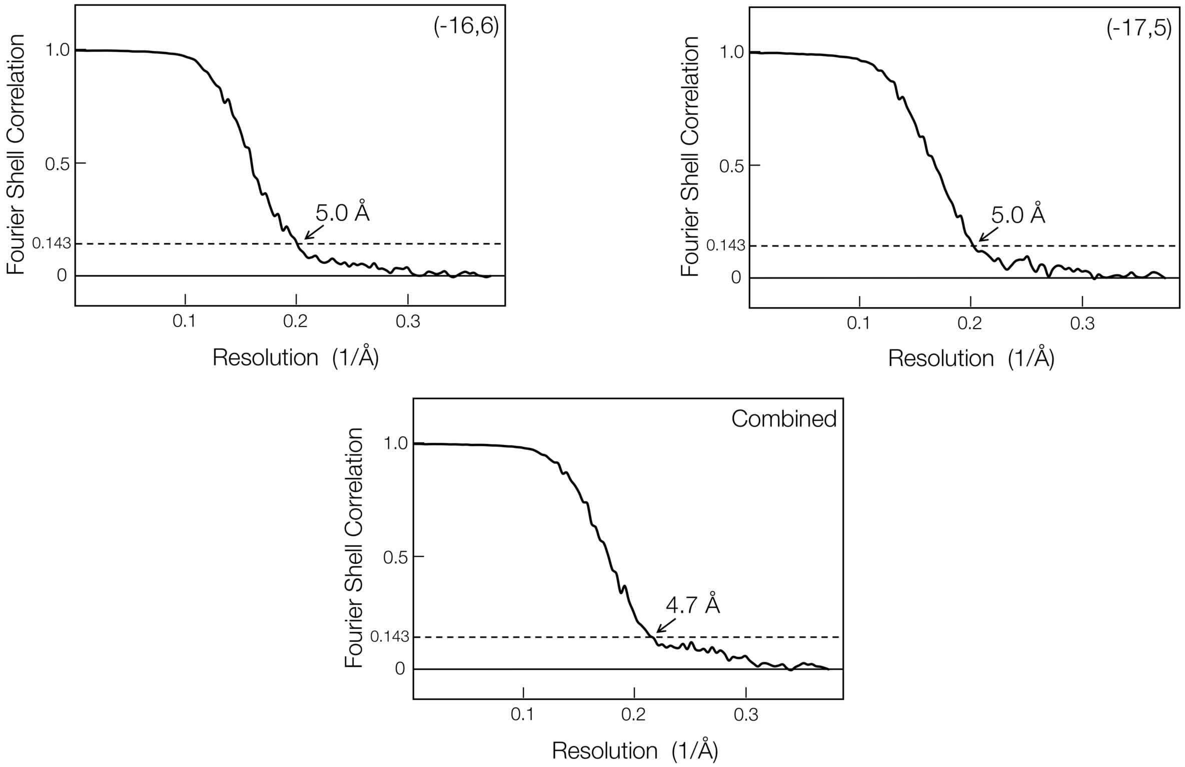

B

Fig. S2. Characterisation of density maps from the (-16,6) and (-17,5) helical families. (*A*) Cross-sections through typical class-average reconstructions in each family. Equivalent (radially aligned) regions around receptors in each class were cut out (red boxes) and averaged. Densities at the base of the receptor most probably arise from the attached (but not helically ordered) protein rapsyn (1, 2), and were not included in the cut-out volumes. (*B*) Fourier shell correlation curves comparing half-set averages from the cut-out volumes in each helical family, and comparing half-set averages from the combined data. The resolutions are 5.0 Å for the family averages and 4.7 Å for the full average, estimated by the FSC = 0.143 threshold.

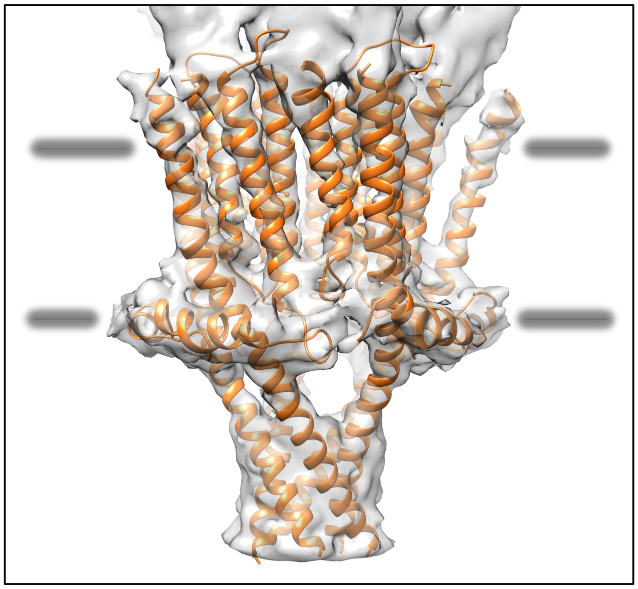

A

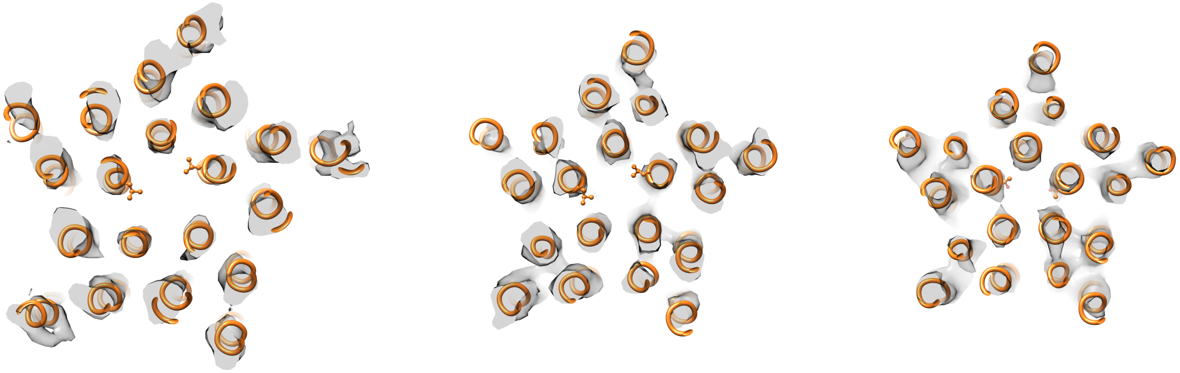

16’ 9’ 2’

B

Fig. S3. Fitting of the membrane portion of the nanodisc-solved structure to the 4.7Å density map. (*A*) Model obtained by real space refinement in *DireX* (3); horizontal bars show locations of peak densities arising from the phospholipid headgroups (4). (*B*) In-plane slabs through densities and superimposed model cutting through the top, middle and bottom portions of the TM helices. These levels are identified by the 16’, 9’ and 2’ pore-facing residues (L, L and T) shown on the alpha subunits. The slabs are about 8 Å thick.

.

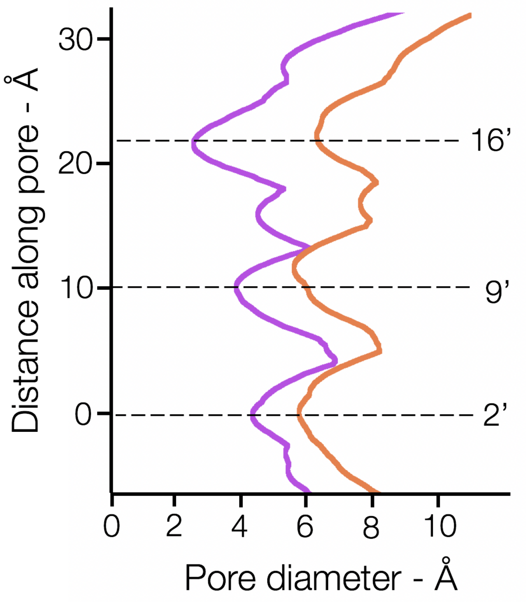

A

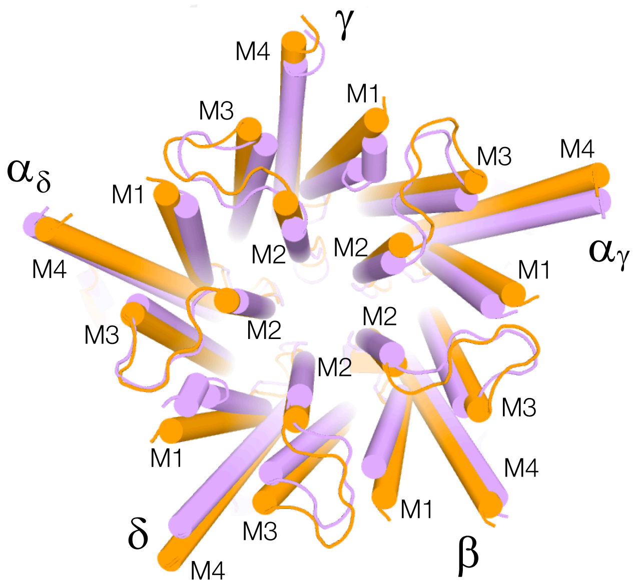

B

Interface	M2-M2 (Å)	M1-M3 (Å)
α_δ / γ	8.8 (6.8)	8.6 (8.2)
γ / α_γ	8.7 (7.4)	8.6 (7.5)
α_γ / β	9.6 (7.0)	9.9 (8.4)
β / δ	9.0 (7.6)	8.9 (7.7)
δ / α_δ	9.2 (7.2)	9.0 (7.8)

C

Fig. S4. Comparison of junctional and nanodisc structures around membrane pore. (*A*) Pore diameters calculated with the program *HOLE* (5). Junctional structure, gold; nanodisc structure, purple. (*B*) Comparison of TM helical arrangements in the two structures. The cylinders representing helices have diameters equivalent to 3 Å. Junctional structure, gold; nanodisc structure, purple. (*C*) Nearest Cα –Cα interface separations at the the level of the phospholipid headgroups; nanodisc values in parentheses.

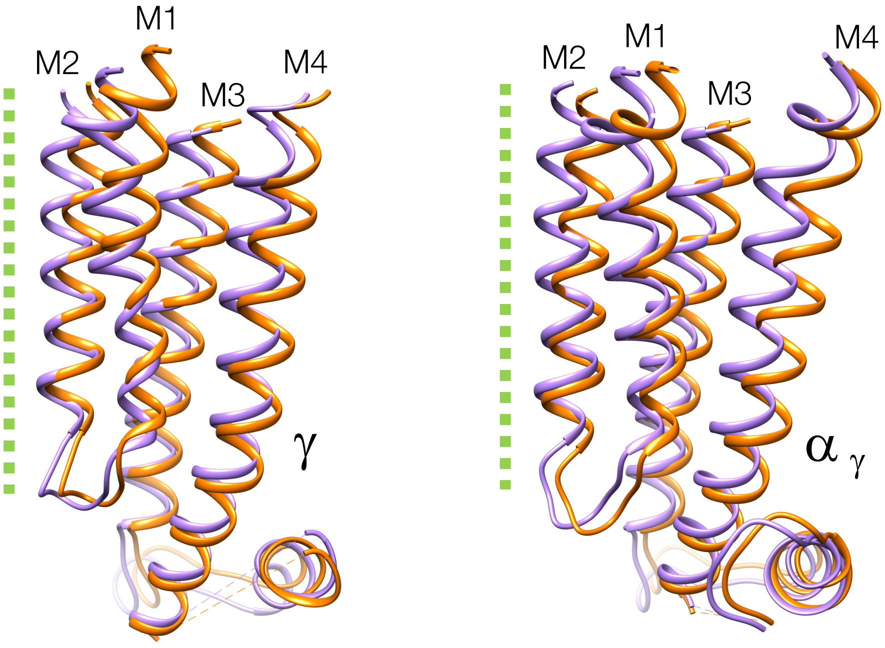

A

Subunit	Current (Å)	Aligned (Å)
γ	2.3	1.3
δ	2.8	1.6
α_δ	1.8	1.1
α_γ	2.1	1.1
β	1.7	1.1

B

Fig. S5. Changes in conformation from nanodisc-solved to junctional structures in the membrane-spanning region. (*A*) These involve internal adjustments in γ and δ, but predominantly rigid-body displacements in α_δ, α_γ, and β. Shown are the changes in γ and α_γ. Nanodisc structure, purple; junctional structure, gold; pore axis, dotted line. (*B*) Root-mean-square deviations (rmsd) between subunit structures (Cα only) before (Current) and after (Aligned) pairwise alignments. The small rmsd values for the aligned α_δ, α_γ and β subunits reflect the predominantly rigid-body nature of their displacements.

Table S1. Details of class-average helical reconstructions contributing to the final density map. Radius at MX varies according to tube diameter; FSC is estimated by the 0.143 threshold.

Accession Code	Tube type	Symmetry	Segments used	Original twist / rise (°/Å)	Imposed twist / rise (°/Å)	Radius at MX (Å)	FSC (Å)
EMD-18785 (6)	(-16,6)	D2	3567	64.760 / 11.52	64.753 / 11.46	271	8.5
EMD-18802 (7)	(-16,6)	D2	3675	64.741 / 11.55	64.753 / 11.46	269	8.6
EMD-18804 (8)	(-16,6)	D2	3752	64.762 / 11.52	64.753 / 11.46	268	8.0
EMD-18805 (9)	(-16,6)	D2	2699	64.762 / 11.62	64.753 / 11.46	271	9.0
EMD-18816 (10)	(-16,6)	D2	3973	64.749 / 11.53	64.753 / 11.46	273	8.8
EMD-18817 (11)	(-16,6)	D2	2541	64.737 / 11.38	64.753 / 11.46	273	8.8
EMD-18823 (12)	(-16,6)	D2	2595	64.727 / 11.45	64.753 / 11.46	271	9.3
EMD-18824 (13)	(-16,6)	D2	3323	64.752 / 11.42	64.753 / 11.46	269	8.7
EMD-18835 (14)	(-16,6)	D2	2190	64.731 / 11.36	64.753 / 11.46	268	9.0
EMD-18831 (15)	(-16,6)	D2	2476	64.751 / 11.59	64.753 / 11.46	265	8.5
EMD-18832 (16)	(-16,6)	D2	2590	64.766 / 11.65	64.753 / 11.46	268	8.6
EMD-18836 (17)	(-16,6)	D2	2230	64.771 / 11.57	64.753 / 11.46	265	8.9
EMD-18837 (18)	(-16,6)	D2	3219	64.732 / 11.50	64.753 / 11.46	267	8.6
EMD-18840 (19)	(-16,6)	D2	3212	64.754 / 11.47	64.753 / 11.46	265	8.3
EMD-18838 (20)	(-16,6)	D2	2083	64.736 / 11.45	64.753 / 11.46	261	8.7
EMD-18843 (21)	(-17,5)	D1	2688	146.980 / 5.82	146.976 / 5.88	265	8.9
EMD-18844 (22)	(-17,5)	D1	3463	146.970 / 5.84	146.976 / 5.88	267	8.4
EMD-18845 (23)	(-17,5)	D1	3110	146.969 / 5.79	146.976 / 5.88	264	8.8
EMD-18846 (24)	(-17,5)	D1	2984	146.989 / 5.93	146.976 / 5.88	264	8.9
EMD-18847 (25)	(-17,5)	D1	4439	146.985 / 5.98	146.976 / 5.88	263	8.5
EMD-18849 (26)	(-17,5)	D1	3409	146.986 / 5.92	146.976 / 5.88	260	8.2
EMD-18850 (27)	(-17,5)	D1	4566	146.975 / 5.92	146.976 / 5.88	263	8.7
EMD-18853 (28)	(-17,5)	D1	2715	146.965 / 5.83	146.976 / 5.88	261	8.9
EMD-18854 (29)	(-17,5)	D1	3095	146.965 / 5.87	146.976 / 5.88	264	9.1
EMD-18855 (30)	(-17,5)	D1	2270	146.964 / 5.88	146.976 / 5.88	261	9.4
EMD-18856 (31)	(-17,5)	D1	2402	146.980 / 5.84	146.976 / 5.88	261	8.7
EMD-18857 (32)	(-17,5)	D1	3110	146.976 / 5.87	146.976 / 5.88	261	8.5
EMD-18858 (33)	(-17,5)	D1	3276	146.976 / 5.86	146.976 / 5.88	264	8.6
EMD-18862 (34)	(-17,5)	D1	3603	146.985 / 5.88	146.976 / 5.88	267	8.8
EMD-18863 (35)	(-17,5)	D1	3150	146.973 / 5.88	146.976 / 5.88	269	9.1
EMD-18865 (36)	(-17,5)	D1	5206	146.975 / 5.90	146.976 / 5.88	265	8.5
EMD-18867 (37)	(-17,5)	D1	4709	146.979 / 5.89	146.976 / 5.88	259	8.2
EMD-18869 (38)	(-17,5)	D1	2766	146.975 / 5.92	146.976 / 5.88	257	9.0
EMD-18870 (39)	(-17,5)	D1	2438	146.973 / 5.85	146.976 / 5.88	256	9.1

References

1. C. Toyoshima, N. Unwin, Three-dimensional structure of the acetylcholine receptor by cryoelectron microscopy and helical image reconstruction. *J. Cell Biol.* **111**, 2623-2635 (1990).

2.. Zuber, N. Unwin, The structure and superorganisation of acetylcholine receptor-rapsyn complexes. *Proc. Natl. Acad. Sci. USA* **110**, 10622-10627 (2013).

3. G. F. Schröder, A.T. Brunger, M. Levitt, Combining efficient conformational sampling with a deformable elastic network model facilitates structure refinement at low resolution. *Structure* **15**, 1630-1641 (2007).

4. N. Unwin, Protein-lipid architecture of a cholinergic postsynaptic membrane. *IUCrJ* **7**, 852-859 (2020).

5. O.S. Smart, J. G. Neduvelil, X. Wang, B.A. Wallace, M.S.P. Sansom, HOLE: a program for the analysis of the pore dimensions of ion channel structural models. *J. Mol. Graph.* **14**, 354–360 (1996).

6. N.Unwin, Influence of lipid bilayer on structure of acetylcholine receptor. Electron Microscopy Data Bank https://www.ebi.ac.uk/emdb/EMD-18785. Deposited 30 October 2023.

7. N.Unwin, Influence of lipid bilayer on structure of acetylcholine receptor. Electron Microscopy Data Bank https://www.ebi.ac.uk/emdb/EMD-18802. Deposited 31 October 2023.

8. N.Unwin, Influence of lipid bilayer on structure of acetylcholine receptor. Electron Microscopy Data Bank https://www.ebi.ac.uk/emdb/EMD-18804. Deposited 1 November 2023.

9. N.Unwin, Influence of lipid bilayer on structure of acetylcholine receptor. Electron Microscopy Data Bank https://www.ebi.ac.uk/emdb/EMD-18805. Deposited 1 November 2023.

10. N.Unwin, Influence of lipid bilayer on structure of acetylcholine receptor. Electron Microscopy Data Bank https://www.ebi.ac.uk/emdb/EMD-18816. Deposited 2 November 2023.

11. N.Unwin, Influence of lipid bilayer on structure of acetylcholine receptor. Electron Microscopy Data Bank https://www.ebi.ac.uk/emdb/EMD-18817. Deposited 2 November 2023.

12. N.Unwin, Influence of lipid bilayer on structure of acetylcholine receptor. Electron Microscopy Data Bank https://www.ebi.ac.uk/emdb/EMD-18823. Deposited 2 November 2023.

13. N.Unwin, Influence of lipid bilayer on structure of acetylcholine receptor. Electron Microscopy Data Bank https://www.ebi.ac.uk/emdb/EMD-18824. Deposited 2 November 2023.

14. N.Unwin, Influence of lipid bilayer on structure of acetylcholine receptor. Electron Microscopy Data Bank https://www.ebi.ac.uk/emdb/EMD-18835. Deposited 3 November 2023.

15. N.Unwin, Influence of lipid bilayer on structure of acetylcholine receptor. Electron Microscopy Data Bank https://www.ebi.ac.uk/emdb/EMD-18831. Deposited 2 November 2023.

16. N.Unwin, Influence of lipid bilayer on structure of acetylcholine receptor. Electron Microscopy Data Bank https://www.ebi.ac.uk/emdb/EMD-18832. Deposited 2 November 2023.

17. N.Unwin, Influence of lipid bilayer on structure of acetylcholine receptor. Electron Microscopy Data Bank https://www.ebi.ac.uk/emdb/EMD-18836. Deposited 3 November 2023.

18. N.Unwin, Influence of lipid bilayer on structure of acetylcholine receptor. Electron Microscopy Data Bank https://www.ebi.ac.uk/emdb/EMD-18837. Deposited 3 November 2023.

19. N.Unwin, Influence of lipid bilayer on structure of acetylcholine receptor. Electron Microscopy Data Bank https://www.ebi.ac.uk/emdb/EMD-18840. Deposited 4 November 2023.

20. N.Unwin, Influence of lipid bilayer on structure of acetylcholine receptor. Electron Microscopy Data Bank https://www.ebi.ac.uk/emdb/EMD-18838. Deposited 3 November 2023.

21. N.Unwin, Influence of lipid bilayer on structure of acetylcholine receptor. Electron Microscopy Data Bank https://www.ebi.ac.uk/emdb/EMD-18843. Deposited 6 November 2023.

22. N.Unwin, Influence of lipid bilayer on structure of acetylcholine receptor. Electron Microscopy Data Bank https://www.ebi.ac.uk/emdb/EMD-18844. Deposited 6 November 2023.

23. N.Unwin, Influence of lipid bilayer on structure of acetylcholine receptor. Electron Microscopy Data Bank https://www.ebi.ac.uk/emdb/EMD-18845. Deposited 6 November 2023.

24. N.Unwin, Influence of lipid bilayer on structure of acetylcholine receptor. Electron Microscopy Data Bank https://www.ebi.ac.uk/emdb/EMD-18846. Deposited 6 November 2023.

25. N.Unwin, Influence of lipid bilayer on structure of acetylcholine receptor. Electron Microscopy Data Bank https://www.ebi.ac.uk/emdb/EMD-18847. Deposited 6 November 2023.

26. N.Unwin, Influence of lipid bilayer on structure of acetylcholine receptor. Electron Microscopy Data Bank https://www.ebi.ac.uk/emdb/EMD-18849. Deposited 6 November 2023.

27. N.Unwin, Influence of lipid bilayer on structure of acetylcholine receptor. Electron Microscopy Data Bank https://www.ebi.ac.uk/emdb/EMD-18850. Deposited 6 November 2023.

28. N.Unwin, Influence of lipid bilayer on structure of acetylcholine receptor. Electron Microscopy Data Bank https://www.ebi.ac.uk/emdb/EMD-18853. Deposited 7 November 2023.

29. N.Unwin, Influence of lipid bilayer on structure of acetylcholine receptor. Electron Microscopy Data Bank https://www.ebi.ac.uk/emdb/EMD-18854. Deposited 7 November 2023.

30. N.Unwin, Influence of lipid bilayer on structure of acetylcholine receptor. Electron Microscopy Data Bank https://www.ebi.ac.uk/emdb/EMD-18855. Deposited 7 November 2023.

31. N.Unwin, Influence of lipid bilayer on structure of acetylcholine receptor. Electron Microscopy Data Bank https://www.ebi.ac.uk/emdb/EMD-18856. Deposited 7 November 2023.

32. N.Unwin, Influence of lipid bilayer on structure of acetylcholine receptor. Electron Microscopy Data Bank https://www.ebi.ac.uk/emdb/EMD-18857. Deposited 7 November 2023.

33. N.Unwin, Influence of lipid bilayer on structure of acetylcholine receptor. Electron Microscopy Data Bank https://www.ebi.ac.uk/emdb/EMD-18858. Deposited 7 November 2023.

34. N.Unwin, Influence of lipid bilayer on structure of acetylcholine receptor. Electron Microscopy Data Bank https://www.ebi.ac.uk/emdb/EMD-18862. Deposited 8 November 2023.

35. N.Unwin, Influence of lipid bilayer on structure of acetylcholine receptor. Electron Microscopy Data Bank https://www.ebi.ac.uk/emdb/EMD-18863. Deposited 8 November 2023.

36. N.Unwin, Influence of lipid bilayer on structure of acetylcholine receptor. Electron Microscopy Data Bank https://www.ebi.ac.uk/emdb/EMD-18865. Deposited 9 November 2023.

37. N.Unwin, Influence of lipid bilayer on structure of acetylcholine receptor. Electron Microscopy Data Bank https://www.ebi.ac.uk/emdb/EMD-18867. Deposited 9 November 2023.

38. N.Unwin, Influence of lipid bilayer on structure of acetylcholine receptor. Electron Microscopy Data Bank https://www.ebi.ac.uk/emdb/EMD-18869. Deposited 9 November 2023.

39. N.Unwin, Influence of lipid bilayer on structure of acetylcholine receptor. Electron Microscopy Data Bank https://www.ebi.ac.uk/emdb/EMD-18870. Deposited 9 November 2023.
